# RETRACTION: Beta3-Adrenergic Receptor Activation Alleviates Cardiac Dysfunction in Cardiac Hypertrophy by Regulating Oxidative Stress

**DOI:** 10.1155/omcl/9832368

**Published:** 2025-10-16

**Authors:** Oxidative Medicine and Cellular Longevity

RETRACTION: M. Zhang, Y. Xu, J. Chen, et al., “Beta3-Adrenergic Receptor Activation Alleviates Cardiac Dysfunction in Cardiac Hypertrophy by Regulating Oxidative Stress,” *Oxidative Medicine and Cellular Longevity* 2021 (2021): 3417242, https://doi.org/10.1155/2021/3417242.

The above article, published online on 04 October 2021 in Wiley Online Library (wileyonlinelibrary.com), has been retracted by agreement between the authors; the journal Chief Editor, Jeannette Vasquez-Vivar; and John Wiley & Sons Ltd. The retraction has been agreed after an investigation of concerns raised by *Hoya camphorifolia* on PubPeer [1], which identified multiple instances of figure duplication:

– The panels showing DAPI treatments in Figure 1b share unexpected similarities with the panels showing DAPI treatments in Figure 4a, which represent a different treatment;
– The panel showing treatment “DAPI – Sham” in Figure 1b and Figure 4a share unexpected similarities with the panel showing “DAPI – MI+SR” in Figure 3a in [2];
– In Figure 5a, the panel showing treatment “DCF – TAC” and the panel showing treatment “DCF - TAC+SR” share unexpected similarities.

The authors have stated that the above concerns were due to inadvertent errors during figure preparation, but have not provided a comprehensive response to all concerns.

An investigation undertaken by the publisher concerning the authenticity of the Figures concluded that the data and conclusions of this article are considered unreliable.
